# UTILIZING PRINCIPLES OF LIFESPAN DEVELOPMENTAL PSYCHOLOGY TO EXAMINE RESILIENCE TO ADVERSITY

**DOI:** 10.1093/geroni/igz038.845

**Published:** 2019-11-08

**Authors:** Frank J Infurna

**Affiliations:** Arizona State University, Tempe, Arizona, United States

## Abstract

Lifespan developmental psychology has many guiding principles. My past, current, and future research has been influenced by the lifespan developmental principles of multidimensionality and multidirectionality and the premise that development is not entirely bound to chronological age, but to historical-, contextual-, non-normative event-, pathology- and mortality-related processes. The first part of my talk will discuss ways I have utilized these principles in my research examining individual’s ability to be resilient to diverse types of adversities (cancer diagnosis, unemployment, and bereavement) and resources they are drawing on to promote more positive outcomes, such as social support. The second part of my talk will focus on future research directions that aim to advance the conceptual and methodological significance of this research. Future directions include explicitly studying individuals in midlife, inclusion of outcomes beyond that of mental health and well-being, such as character strengths, and longitudinal research designs that assess people more frequently.

